# Cardiac structure and function after revascularization versus medical therapy for renal artery stenosis: the ASTRAL heart echocardiographic sub-study

**DOI:** 10.1186/s12882-019-1406-y

**Published:** 2019-06-14

**Authors:** Darren Green, Diana Vassallo, Kelly Handley, Natalie Ives, Keith Wheatley, Constantina Chrysochou, Janet Hegarty, Julian Wright, Jon Moss, Rajan K. Patel, Chris Deighan, John Webster, Peter Rowe, Sue Carr, Jenny Cross, Jamie O’Driscoll, Raj Sharma, Patrick Mark, Philip A. Kalra

**Affiliations:** 1 Department of Renal Medicine, Salford Royal NHS Foundation Trust, Stott Lane, Salford, M6 8HD UK; 2 University of Birmingham, Birmingham, UK; 3 Manchester Foundation Trust, Manchester, UK; 4 Western Infirmary, Glasgow, UK; 5 Glasgow Royal Infirmary, Glasgow, UK; 6 Aberdeen Royal Infirmary, Aberdeen, UK; 7 Derriford Hospital, Plymouth, UK; 8 University Hospitals of Leicester, Leicester, UK; 9 Royal Free Hospital, London, UK; 10 St George’s Hospital, London, UK; 11 Institute of Cardiovascular and Medical Science, University of Glasgow, Glasgow, UK

**Keywords:** Renal artery stenosis, Revascularization, Echocardiography, Left ventricular hypertrophy, Randomized controlled trial

## Abstract

**Background:**

The ASTRAL trial showed no difference in clinical outcomes between medical therapy and revascularization for atherosclerotic renal vascular disease (ARVD). Here we report a sub-study using echocardiography to assess differences in cardiac structure and function at 12 months.

**Methods:**

ASTRAL patients from 7 participating centres underwent echocardiography at baseline and 12 months after randomisation. Changes in left ventricular ejection fraction (LVEF), left ventricular mass (LVM), left atrial diameter (LAD), aortic root diameter (AoRD), E:A, and E deceleration time (EDT) were compared between study arms. Analyses were performed using t-tests and multivariate linear regression.

**Results:**

Ninety two patients were included (50 medical versus 42 revascularization). There was no difference between arms in any baseline echocardiographic parameter. Comparisons of longitudinal changes in echocardiographic measurements were: δLVEF medical 0.8 ± 8.7% versus revascularization − 2.8 ± 6.8% (*p* = 0.05), δLVM − 2.9 ± 33 versus − 1.7 ± 39 g (*p* = 0.9), δLAD 0.1 ± 0.4 versus 0.01 ± 0.5 cm (*p* = 0.3), δAoRD 0.002 ± 0.3 versus 0.06 ± 0.3 cm (*p* = 0.4), δE:A − 0.0005 ± 0.6 versus 0.03 ± 0.7 (*p* = 0.8), δEDT − 1.1 ± 55.5 versus − 9.0 ± 70.2 ms (*p* = 0.6). In multivariate models, there were no differences between treatment groups for any parameter at 12 months. Likewise, change in blood pressure did not differ between arms (mean δsystolic blood pressure medical 0 mmHg [range − 56 to + 54], revascularization − 3 mmHg [− 61 to + 59], *p* = 0.60).

**Conclusions:**

This sub-study did not show any significant differences in cardiac structure and function accompanying renal revascularization in ASTRAL. Limitations include the small sample size, the relative insensitivity of echocardiography, and the fact that a large proportion of ASTRAL patient population had only modest renal artery stenosis as described in the main study.

## Background

The presence of atherosclerotic renovascular disease (ARVD) in patients with chronic kidney disease (CKD) presents an increased risk of cardiovascular morbidity compared to other causes of CKD. ARVD is typically associated with extensive extra-renal atherosclerosis and significant cardiovascular comorbidities; 91% of ARVD patients have hypertension, 38% have clinical heart failure, and 67% have coronary artery disease [1, 2].

As a result of these overlapping cardiovascular risk factors, cardiac structural remodelling is almost ubiquitous in ARVD. At least three-quarters of ARVD patients have left ventricular hypertrophy and diastolic dysfunction on echocardiography [3]. Patients with ARVD have 58% greater LV mass than eGFR matched CKD controls [3], and likewise greater LV mass than non-CKD hypertension controls [4].

Two large clinical trials failed to show clinical benefit of renal artery revascularization when used as first line therapy for ARVD. The primary outcome measure of the ASTRAL (Angioplasty and Stenting for Renal Artery Lesions) trial was longitudinal change in renal function, while that of the CORAL (Cardiovascular Outcomes in Renal Atherosclerotic Lesions) trial was a composite of death or major morbidity from cardiovascular or renal causes [5, 6].

Although revascularization does not benefit most patients, there are many documented cases of significant clinical and cardiac structural changes soon after intervention [7, 8]. These suggest that revascularization may be of benefit either in specific ARVD sub-groups, or that patients undergo significant cardiac remodelling after renal artery revascularization that does not manifest as a reduction in the acute events recorded as end-points in clinical trials. Whilst reporting bias is certain in these reports, flash pulmonary oedema is the only clinical scenario that has attracted Class I recommendation for revascularization [9, 10].

Flash pulmonary oedema is however the presenting feature in only 12% of cases of ARVD [11], meaning that the majority of patients with ARVD do not appear to suffer acute decompensation of heart failure despite underlying cardiac remodelling. In these patients, any change in cardiac structure and function that may occur as a result of revascularization might not manifest as a reduction in the acute cardiovascular events measured by ASTRAL and CORAL. It is therefore reasonable to hypothesise that patients with ARVD and abnormal hearts who do not present with flash pulmonary oedema may show sub-clinical improvements in cardiac structure and function after revascularization compared to standard medical therapy [12, 13].

Here we present findings from an echocardiographic sub-study of the ASTRAL trial. The primary aim of this study was to evaluate whether the addition of renal artery revascularization to standard medical therapy led to improvements in the cardiac structural and functional abnormalities associated with ARVD, as found on echocardiography, compared to standard medical therapy alone. A secondary aim was to quantitatively describe the natural progression of cardiac abnormalities in an ARVD cohort, irrespective of therapy, and specifically in patients for whom revascularization is not already indicated.

## Methods

The detailed method of patient selection and intervention for ASTRAL has previously been published [5]. In summary, ASTRAL was a multicentre, non-blinded clinical trial comparing outcomes in ARVD between patients randomised to receive either medical therapy or medical therapy plus percutaneous renal artery revascularization **(**Clinical Trials Registration ISRCTN59586944). Ethical approval for ASTRAL was granted by the West Midlands Multicentre Research Ethics Committee, UK and the ethics committee relevant to each individual participating study centre. Ethical permission for the echocardiographic sub-study was obtained separately. Support for the main ASTRAL trial was received from the Medical Research Council UK, Kidney Research UK, and Medtronic.

### Patient selection

Patients were eligible for ASTRAL if they had at least one renal artery with an atherosclerotic lesion suitable for percutaneous revascularization and their managing clinician was not convinced that revascularization was essential. Patients were excluded if revascularization was already indicated as per guidelines, if the patient had undergone previous revascularization, or if the stenosis was not atherosclerotic in origin. No patients on haemodialysis were entered into the study, and because flash pulmonary oedema is a long-standing agreed indication for revascularization in ARVD, such patients were not recruited to ASTRAL.

Patients from 7 centres participating in ASTRAL were approached to take part in this cardiac sub-study (Salford Royal Hospital, Manchester Royal Infirmary, Royal Free London, Aberdeen Royal Infirmary, Derriford Hospital Plymouth, Glasgow Royal Infirmary, University Hospitals of Leicester). Patients were randomised 1:1 into the ASTRAL study to either revascularization or no revascularization, and this also determined the randomised treatment allocation of patients in the sub-study. No specific exclusion criteria were applied to the echocardiographic sub-study, and every patient provided signed informed consent.

### Echocardiography protocol

Patients underwent a full cross-sectional transthoracic echocardiogram within 6 weeks of enrolment and, if randomised to revascularization, before they underwent the procedure. A second transthoracic echocardiography was obtained 1 year later. Scans were performed locally at each participating centre by technicians accredited for transthoracic echocardiography by the British Society for Echocardiography. Interpretation was performed by a single imaging consultant cardiologist blind to the date of each scan and the participant’s randomised treatment allocation.

All image acquisitions and measurements were performed as recommended by the American Society of Echocardiography [14] and have been described previously [15]. In brief, left ventricular end diastolic diameter (LVEDD), LV end systolic diameter (LVESD), interventricular and LV posterior wall thickness at end diastole were measured from parasternal long-axis M mode recordings of the LV. The modified biplane Simpson’s rule was used to determine the LV ejection fraction, with measurements averaged over three cardiac cycles. Pulsed wave Doppler recordings at the mitral valve leaflet tips in the apical four-chamber view were used to record transmitral flow. Peak velocity of early filling (E), peak velocity of atrial filling (A), the E/A ratio, E-deceleration time (ms) and isovolumetric relaxation time (IVRT) were measured. LV mass was calculated using the Devereux Formula. LV regional wall motion was analysed visually using the standard 17-segment model for qualitative analysis and wall motion was scored on a 4-point scale (1 = normal wall motion, 2 = hypokinesis, 3 = akinesis, and 4 = dyskinesis). The wall motion score index was calculated as an average of the individual wall motion scores of each visualised segment. Echocardiographic evaluation of the aorta was performed in the parasternal long-axis and suprasternal view with measures recorded at the tubular ascending aorta. The severity of valve disease was determined by the physician’s visual assessment and graded as mild, moderate or severe [16].

### Sample size

Sample size calculation was based on left ventricular end diastolic diameter (LVEDD), one of the more sensitive parameters of change in cardiac function over time, or after intervention, and based upon change in LVEDD in a previous study of 79 ARVD patients (mean LVEDD 5.37 ± 0.95 cm). To detect a 0.6 cm (SD 0.95) difference between revascularization and control groups at a power of 80%, 41 patients would be needed for each group with an alpha of 0.05. At 90% power, patient numbers would be 54 per group.

### Statistical analysis

The main end-points were change from baseline to follow up in each of the echocardiographic parameters listed above. The two treatment groups of medical therapy alone and medical therapy plus revascularization were compared using 2-sample t-tests. Multivariate linear regression models were then constructed to consider the effects of other pre-specified clinically relevant baseline variables on each of the echocardiographic parameters at 12 months. Alongside treatment group, the following variables were included in each model: age, presence of diabetes, history of coronary heart disease, systolic blood pressure, diastolic blood pressure, degree of stenosis to most affected kidney, renal function using eGFR, prescription of beta-blockers and renin angiotensin blockade and baseline ventricular measurement. A *p*-value of < 0.05 was considered statistically significant. All analyses were performed using SAS version 9.1 (SAS Institute, Cary, NC, USA).

## Results

There were 92 patients included in the study (50 medical, 42 revascularization). The two arms were broadly comparable: the age in the medical arm was 71 years (range 51–86) compared with 70 (53–86) years in the revascularization arm (*p* = 0.9). There was no difference in baseline eGFR, calculated using the Cockroft-Gault equation: 43.0 (17.0–79.7) ml/min versus 44.7 (15.4–89.8) ml/min (*p* = 0.65). Similarly, there was no difference in systolic blood pressure: 152 (90–220) mmHg versus 146 (103–196) mmHg (*p* = 0.36). A full comparison of baseline characteristics is found in Table 1. The only difference between the arms was that patients in the medical therapy group were more likely to be on lipid lowering therapy than those in the revascularization group (93% versus 78%, *p* = 0.04). However, serum cholesterol was no different: 4.5 (2.8–7.9) mmol/L versus 4.3 (2.3–6.5) mmol/L, *p* = 0.43.

**Table 1:** Baseline characteristics comparing medical therapy versus revascularization patients

	Medical (*N* = 50)	Revascularization (*N* = 42)	p
Demographic			
Mean age (range) – years	71 (51–86)	70 (53–86)	0.90
Male sex – no. (%)	34 (68%)	33 (79%)	0.26
Clinical			
Smoking status – no./total no (%)			
Current smoker	11/46 (24%)	8/36 (22%)	0.86
Former smoker	24/46 (52%)	19/36 (53%)	0.96
Coexisting conditions – no./total no (%)			
Diabetes	15/47 (32%)	13/37 (35%)	0.76
Coronary heart disease	25/47 (53%)	22/37 (59%)	0.57
Peripheral vascular disease	20/46 (43%)	17/37 (46%)	0.82
Stroke	14/47 (30%)	8/37 (22%)	0.40
Need for dialysis	0	0	–
Renal Function			
Mean serum creatinine (range) – μmol/litre	165 (64–326)	170 (68–534)	0.76
Mean eGFR (range) – ml/min	43.0 (17.0–79.7)	44.7 (15.4–89.8)	0.65
Mean proteinuria (range) – g/day	0.45 (0.01–2.20)	0.42 (0.00–1.70)	0.90
Related Measures			
Mean systolic BP (range) – mmHg	152 (90–220)	146 (103–196)	0.36
Mean diastolic BP (range) – mmHg	74 (57–97)	74 (45–102)	0.93
Mean total cholesterol (range) – mmol/litre	4.5 (2.8–7.9)	4.3 (2.3–6.5)	0.43
Renal Physiology			
Mean stenosis (range) – %	73.6 (50–99)	71.2 (50–95)	0.41
Severity of stenosis- no (%) 50–70%	26 (52%)	25 (60%)	0.47
> 70%	24 (48%)	17 (40%)	–
Mean bipolar kidney length (range) – cm	9.6 (7.0–11.9)	9.7 (6.4–12.5)	0.70
Use of Concomitant medication			
Mean no. of antihypertensive drugs (range)	3.0 (1–5)	2.9 (1–5)	0.66
Any antiplatelet drug – no./total no. (%)	38/47 (81%)	31/37 (84%)	0.73
Lipid lowering therapy – no./total no. (%)	43/46 (93%)	29/37 (78%)	0.04
Warfarin – no./total no. (%)	2/47 (4%)	5/36 (14%)	0.12

Key: *BP* blood pressure, *eGFR* estimated glomerular filtration rate

The baseline echocardiographic measurements were all comparable between groups, with none showing a statistically significant difference. Systolic function was well preserved overall (54 ± 10%). The complete list of baseline echocardiographic variables is found in Table 2. This table also shows the change in each parameter between baseline and follow up. There was no difference between the medical and revascularization arms for longitudinal change in any echocardiographic parameter on univariate analysis. These are detailed in full in Table 2. LVEF was closest to showing a statistical difference (medical 0.8 ± 8.7% versus revascularization − 2.8 ± 6.8%, *p* = 0.05).

**Table 2:** Comparison of echocardiographic parameters at baseline between trial arms and comparison of the changes in measurement on the follow up scans compared to baseline

	Baseline		Change at follow up		
	Medical	Revasc	Medical	Revasc	p
Aortic root diameter (cm)	3.1 ± 0.4	3.1 ± 0.4	0.002 ± 0.3	0.06 ± 0.3	0.4
LVOT velocity (m/s)	1.2 ± 0.2	1.2 ± 0.2	−0.02 ± 0.1	−0.03 ± 0.1	0.7
LV ejection fraction (%)	53.8 ± 10.5	53.6 ± 9.2	0.8 ± 8.7	−2.8 ± 6.8	0.05
Left atrial diameter (cm)	3.8 ± 0.5	3.9 ± 0.5	0.1 ± 0.4	0.01 ± 0.5	0.3
LVESD (cm)	2.8 ± 0.6	2.8 ± 0.5	− 0.07 ± 0.4	0.1 ± 0.6	0.1
LVEDD (cm)	4.9 ± 0.4	4.9 ± 0.4	− 0.08 ± 0.4	− 0.07 ± 0.4	0.9
Left ventricular mass (g)	203 ± 37	202 ± 34	− 2.9 ± 33	−1.7 ± 39	0.9
Relative wall thickness	0.45 ± 0.08	0.44 ± 0.06	0.01 ± 0.06	0.01 ± 0.06	0.98
E:A	1.1 ± 0.6	1.1 ± 0.6	− 0.0005 ± 0.6	0.03 ± 0.7	0.8
E deceleration time (ms)	206 ± 59	202 ± 66	− 1.1 ± 55.5	−9.0 ± 70.2	0.6

Data are mean ± standard deviation

Key: *revasc* revascularization arm, *LVOT* left ventricular outflow tract, *LV* left ventricular, *ESD* end systolic diameter, *EDD* end diastolic diameter, *n/s* not significant

Importantly, allied to this, there was no change in blood pressure between arms during follow up. In the medical arm, the mean systolic blood pressure change during follow up was 0 mmHg (range − 56 to + 54 mmHg). This compared with a mean change of − 3 (− 61 to + 59) mmHg in the revascularization arm (*p* = 0.60). For diastolic blood pressure, the respective changes were 0 (− 27 to + 80) mmHg versus − 4 (− 41 to + 20) mmHg (*p* = 0.27).

Table 3 summarises the output of the multivariate regression models for each of the echocardiographic parameters. Treatment modality did not alter outcome for any echocardiographic parameter including left ventricular mass, volume and ejection fraction. Other baseline factors did demonstrate significance, particularly medication although no drugs were consistently significant across all echocardiographic parameters. Beta-blockade was associated with better aortic root diameter at follow up, but higher left ventricular mass and relative wall thickness. ACE inhibitor use was also associated with higher relative wall thickness.

**Table 3:** Factors associated with echocardiographic measurements at follow up based on multivariate analysis

Measurement	Significant factors	Estimate	Standard Error	95% CI	p
AoRD	Baseline AoRD	0.83	0.08	0.66 to 0.99	< 0.0001
	Beta-blocker use - No	0.20	0.07	0.07 to 0.34	0.004
LVOT velocity	Baseline LVOT velocity	0.73	0.08	0.58 to 0.88	< 0.0001
LVEF	Baseline LVEF	0.61	0.08	0.45 to 0.77	< 0.0001
LA diameter	Baseline LA diameter	0.88	0.11	0.67 to 1.10	< 0.0001
LVESD	Baseline LVESD	0.51	0.08	0.34 to 0.67	< 0.0001
LVEDD	Baseline LVEDD	0.52	0.08	0.35 to 0.68	< 0.0001
	Age	−0.01	0.005	−0.02 to − 0.0003	0.04
LV mass	Baseline LV mass	0.60	0.09	0.42 to 0.77	< 0.0001
	Coronary heart disease - No	20.1	6.54	7.02 to 33.19	0.003
	eGFR	−0.49	0.20	−0.89 to − 0.09	0.02
	ACE inhibitor use - No	−13.2	6.26	−25.74 to −0.68	0.04
E:A	Baseline E:A	0.38	0.11	0.15 to 0.61	0.002
	Diabetes - No	−0.30	0.14	− 0.58 to − 0.01	0.04
EDT	Baseline EDT	0.40	0.10	0.20 to 0.59	0.0001
	Age	1.99	0.95	0.08 to 3.89	0.04
	eGFR	0.93	0.38	0.17 to 1.68	0.01
RWT	Baseline RWT	0.68	0.08	0.51 to 0.84	< 0.001
	Beta-Blocker use - No	−0.02	0.01	−0.04 to −0.001	0.04
	ACE inhibitor use - No	−0.03	0.01	−0.06 to − 0.01	0.003

Key: *LVOT* left ventricular outflow tract, *LVEF* left ventricular ejection fraction, *LA* left atrium, *LVESD* left ventricular end systolic diameter, *LVEDD* left ventricular end diastolic diameter, *EDT* E wave deceleration time, *RWT* relative wall thickness, *CI* confidence intervals, *eGFR* estimated glomerular filtration rate, *AoRD* aortic root diameter

Each model included the following variables: treatment group, age, presence of diabetes, history of coronary heart disease, systolic blood pressure, diastolic blood pressure, degree of stenosis to most affected kidney, renal function using eGFR, prescription of beta-blockers and renin angiotensin blockade and the baseline ventricular measurement

When comparing all follow up scans with all baseline scans, there was no overall significant difference between any individual parameter using paired t-test: LVEF baseline = 54 ± 10% versus LVEF at follow up = 53 ± 9%, *p* = 0.40; left atrial diameter = 4.0 ± 0.5 cm versus 4.0 ± 0.6 cm, *p* = 0.19; LVEDD = 4.9 ± 0.4 versus 4.8 ± 0.4, *p* = 0.07, LV mass = 206 ± 37 g versus 204 ± 37 g, *p* = 0.65. Although the overall pattern was of no change, individual cases did show deterioration or improvement in structure or function on echocardiography, albeit without a difference between the treatment arms as outlined above. The range of change in LVEF was − 19 to + 27% (median 0%), for left atrial diameter was − 1.1 cm to + 1.4 cm (median 0 cm), for LVEDD was − 0.9 cm to + 1.0 cm (median − 0.1 cm), and for LV mass was -101 g to + 129 g (median 0 g).

**Fig. 1:**
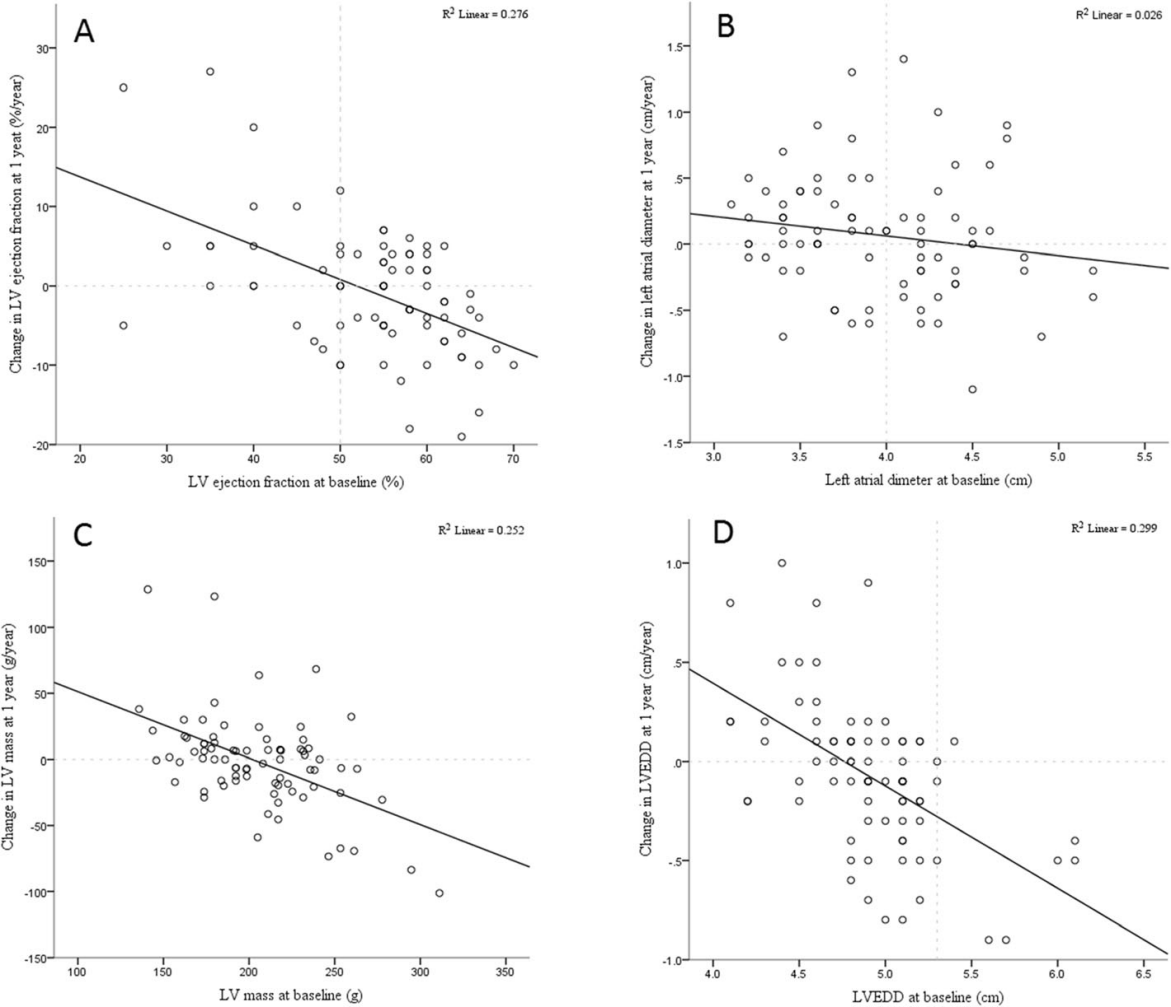
Correlation between change in echocardiographic measurements at 1 year compared to baseline measurement for **a**) left ventricular (LV) ejection fraction, **b**) left atrial diameter, **c**) left ventricular mass, **d**) left ventricular end diastolic diameter (LVEDD)

Figure 1 shows the correlation between baseline measurements of LVEF, left atrial diameter, LVEDD and LV mass, and the change in these parameters at follow up compared to baseline. For LVEF, 54% of patients with preserved systolic function at baseline (LVEF≥50%) had a worse LVEF at follow up. This compared with 24% of patients who had pre-existing systolic impairment at baseline (LVEF< 50%). The correlation coefficient between baseline LVEF and change in LVEF was − 0.53, *p* < 0.01.

In a between group comparison of dLVEF between those with preserved baseline LVEF (≥50% versus those with reduced LVEF (< 50%) at baseline, the mean change in the preserved function group was − 2.4 ± 6.4 mL versus + 5.2 ± 10.5 mL in the reduced function group (*p* = 0.01). The distribution was parametric.

For LA diameter, 54% of patients with a dilated left atrium (diameter > 4 cm) at baseline showed an improvement in diameter at follow up. The figure for baseline normal left atrium was 25%. For change in left atrial diameter against baseline reading, the correlation coefficient was − 0.16, *p* = 0.16. Only 5 patients had LVEDD > 5.3 cm at baseline but all of these showed improvement at follow up. Change in left ventricular mass followed a similar pattern to that of LVEF (Fig. 1).

In a between group comparison of change in left atrial diameter between those with normal baseline diameter (< 4.0 cm) versus those with increased diameter (≥4.0 cm), the mean change in the normal group was + 0.1 ± 0.4 cm versus 0.0 ± 0.5 cm in the dilated group (*p* = 0.201).

## Discussion

This sub-study of a randomised clinical trial included 92 patients with ARVD and did not show any difference in longitudinal change in echocardiographic parameters between patients treated with renal artery revascularization versus those given medical therapy alone. This is consistent with the cardiovascular end point findings of the main ASTRAL [5] (hazard ratio for cardiovascular events 0.94, 95% CI 0.75–1.19, *p* = 0.61) and CORAL [6] studies (hazard ratio for composite primary end-point 0.94; 95% CI 0.76–1.17; *p* = 0.58).

Our findings are consistent with two other randomised clinical trials. A recent parallel ASTRAL sub-study investigated the effects of revascularization on cardiac structure and function using cardiac magnetic resonance imaging in 18 patients treated exclusively medically and 23 patients who underwent revascularization in addition to treatment with medical therapy. Although slight improvements in cardiac structural parameters were seen in both groups at 12 months follow-up, there were no significant differences between groups (change in LVM in medical versus revascularized group: − 5.4 g versus − 6.3 g, *p* = 0.8) [17]. The stenting of Renal Artery Stenosis in Coronary Artery Disease (RAS-CAD) study is another randomised trial that explored the effect of revascularization on LVMI in ARVD patients with mean renal artery stenosis < 70%, mean eGFR > 60.0 ml/min/1.73m^2^ and well-controlled blood pressure; patients in both arms were established on multi-targeted medical therapy. Revascularization was shown to have no additional impact on cardiac structure and blood pressure control beyond optimal medical therapy. However, there was again significant equivalent improvement in blood pressure control and LVMI in both revascularized (*n* = 43) and non-revascularized patients (*n* = 41) [18].

In contrast, in our study there was no overall difference in parameters at follow up compared to baseline. Previous small non-randomised studies had demonstrated improvement in left ventricular mass in patients with renovascular hypertension or haemodynamically significant stenosis. In two of these studies, this was shown to correlate with improvement in blood pressure control [19, 20]. In ASTRAL, including this sub-study, there was no difference in blood pressure control between treatment arms. This likely explains the lack of any difference in δLVM seen here.

Although a lack of longitudinal improvement was noted in this study, which differs from the results of ASTRAL cardiac magnetic resonance imaging sub-study and RAS-CAD [17, 18], this is not surprising given the relative insensitivity of transthoracic echocardiography for certain measurements (e.g. LV mass) compared to cardiac magnetic resonance imaging. Left ventricular mass was not adjusted for height and weight due to missing data hence this may also have contributed to reduced accuracy. Regardless, the outcome measurement is change in cardiac mass over time for each individual patient. Given this, it is unlikely that there would be any additional benefit to indexing these measurements against body surface area or height.

In view of the lack of measurable longitudinal changes observed in our study, we support the current view that optimised medical therapy is as effective as revascularization for routine ARVD therapy, except in those patients for whom revascularization is already indicated. Indeed, in the current study we provided examples of patients who showed definite improvement or stabilisation in echocardiographic parameters at 1 year, and likewise some with evidence of deterioration and this is of clinical importance. This comment is made notwithstanding the possibility that the longitudinal changes seen represent regression to the mean in a population with a broad baseline measurement in echocardiographic descriptors.

Our results suggest a weak inverse correlation between baseline function and improvement at 1 year, in keeping with results from observational studies showing that revascularization only appears to be of benefit in a small subset of ‘high-risk’ patients [11, 21, 22]. Future research efforts are likely to be directed towards the timely identification of these individuals through risk stratification techniques with the aim of improving patient selection for revascularization.

In our study, beta-blockade was associated with reduction in aortic root diameter. In light of the predominance of diastolic dysfunction in patients with ARVD [3], this is presumed to be a function of blood pressure reduction and consequent improved diastolic function, though we have no direct evidence of causation from this trial. Beta-blockers have been consistently shown to prevent adverse cardiac remodelling and optimise clinical outcomes in patients with reduced ejection fractions [23–25], although there is a concern that in patients with heart failure with preserved ejection fraction (HFpEF) beta-blockers can precipitate a negative chronotropic effect and reduced exercise tolerance [26]. Their value in patients with non-ischaemic heart failure with preserved ejection fraction has however not been studied. Given the upregulated sympathetic drive and the high rate of co-existent cardiovascular disease in ARVD patients [1], our findings point towards the use of such therapy in these patients. Indeed, a recent retrospective analysis performed on 529 patients with ARVD showed that over a median follow-up period of 3.8 years, beta-blockade was associated with reduced risk of death (relative risk 0.52 [95% CI 0.31–0.89], *p* = 0.02) and nonfatal cardiovascular events (relative risk 0.74 [95% CI 0.60–0.90], *p* = 0.003) [27].

In the context of non-ARVD patients with hypertension and diastolic dysfunction without a clinical diagnosis of heart failure, angiotensin-receptor blockers have been shown to reduce left ventricular mass and function and improve exercise tolerance; the changes in RWT observed in this study did not mirror this [28–30], but renin-angiotensin blockade has been shown to be associated with improved clinical outcomes in observational studies carried out in patients with ARVD [31, 32]. Given the existing knowledge of the pathophysiology of diastolic dysfunction in these patients, renin-angiotensin blockade remains part of the standard of care for these patients.

This study has limitations. Due to the slightly lower than expected number of patients, the study had 80%, rather than 90%, power to detect differences of the anticipated magnitude. Finally, as mentioned above, an important consideration is that some patients recruited into this study may have had clinical and haemodynamically insignificant RAS and so these results are not necessarily generalizable to all patients with ARVD, particularly those with severe renal artery stenosis presenting with heart failure and other high-risk clinical phenotypes [11].

## Conclusion

In this ASTRAL sub-study, there was no significant difference in longitudinal change in echocardiographic parameters between revascularized and non-revascularized patients, nor between baseline and follow-up scans in the study population as a whole. These results are consistent with the neutral renal, cardiovascular and mortality end-points observed in the main ASTRAL trial and in the subsequent CORAL trial.

## Data Availability

The data that support the findings of this study are available from the Birmingham Clinical Trials Unit and Salford Royal NHS Foundation Trust but restrictions apply to the availability of these data, which were used under license for the current study, and so are not publicly available. Data are however available from the authors upon reasonable request and with permission of the Birmingham Clinical Trials Unit and Salford Royal Research and Development Department.

## Abbreviations

A: Peak velocity of atrial filling
ACE: Angiotensin converting enzyme
AoRD: Aortic root diameter
ARVD: Atherosclerotic renovascular disease
ASTRAL: Angioplasty and Stenting for Renal Artery Lesions
CI: Confidence intervals
CKD: Chronic kidney disease
CORAL: Cardiovascular Outcomes in Renal Atherosclerotic Lesions
E: Peak velocity of early filling
EDT: E wave deceleration time
eGFR: Estimated glomerular filtration rate.
FGF-23: Fibroblast growth factor-23
IVRT: Isovolumetric relaxation time
LV: Left ventricle
LVEDD: Left ventricular end diastolic diameter,
LVEF: Left ventricular ejection fraction
LVESD: Left ventricular end systolic diameter,
LVM: Left ventricular mass
LVOT: Left ventricular outflow tract
PDGF: Platelet derived growth factor
RAS-CAD: Renal artery stenosis coronary artery disease
RWT: Relative wall thickness
TGF-β: Transforming growth factor beta
